# The Effect of Sodium Valproate on the Glioblastoma U87 Cell Line Tumor Development on the Chicken Embryo Chorioallantoic Membrane and on EZH2 and p53 Expression

**DOI:** 10.1155/2017/6326053

**Published:** 2017-05-31

**Authors:** Dovilė Kavaliauskaitė, Donatas Stakišaitis, Justė Martinkutė, Lina Šlekienė, Arūnas Kazlauskas, Ingrida Balnytė, Vaiva Lesauskaitė, Angelija Valančiūtė

**Affiliations:** ^1^Department of Histology and Embryology, Lithuanian University of Health Sciences, Mickeviciaus Str., LT-44307 Kaunas, Lithuania; ^2^Laboratory of Cancer Epidemiology, National Cancer Institute, Santariškių Str. 1, LT-08660 Vilnius, Lithuania; ^3^Lithuanian University of Health Sciences, Neuroscience Institute, Laboratory of Neurooncology and Genetics, Eivenių 4, LT-50161 Kaunas, Lithuania; ^4^Lithuanian University of Health Sciences, Institute of Cardiology, Laboratory of Molecular Cardiology, Suklilėlių 17, LT-50161 Kaunas, Lithuania

## Abstract

Literature data support evidences that glioblastoma (GBM) patients experience prolonged survival due to sodium valproate (NaVP) treatment. The study assessed the human GBM cell U87 xenograft studied in the chicken embryo chorioallantoic membrane (CAM) model evaluating NaVP effect on tumor. Three groups of tumors (each* n* = 10) were studied: nontreated, treated with 4 mM, and treated with 8 mM of NaVP. The majority of tumors without NaVP treatment during tumor growth destroyed the chorionic epithelium, invaded the mesenchyme, and induced angiogenesis. Incidence of tumor formation on CAM without invasion into the mesenchyme was higher when U87 cells were treated with NaVP; the effect significantly increased with NaVP concentration. Treatment with 8 mM of NaVP did not show clear dynamics of tumor growth during 5 days; at the same time, the angiogenesis failed. With a strong staining of EZH2, p53 in tumors without NaVP treatment was found, and NaVP significantly decreased the expression of EZH2- and p53-positive cells; the effect was significantly higher at its 8 mM concentration. NaVP has a function in blocking the growth, invasion, and angiogenesis of tumor in the CAM model; tumor growth interferes with EZH2 and p53 molecular pathways, supporting the NaVP potential in GBM therapy.

## 1. Introduction

Glioblastoma multiforme (GBM) is the most frequent, highly recurrent, and rapidly progressing type of astrocytic brain tumor in adults [1]. Epileptic seizures occur in approximately 50% of GBM patients [2, 3]. Sodium valproate (NaVP) is an authorized medicinal product for the treatment of epileptic seizure, migraine, neuralgia, and bipolar disorder [4, 5]. Glioma patients with a history of seizures have a better prognosis than patients without seizures and it has been reported that this phenomenon could be related to the NaVP used for seizure prophylaxis or treatment. The meta-analysis of studies data also supports the evidence that glioblastoma patients experience prolonged survival due to NaVP treatment [6, 7].

The mechanisms of NaVP without an antiepileptic activity are the known inhibitor of histone deacetylase [4]. It has an anticancer effect in several human GBM cell lines [8]. Preclinical studies have suggested that NaVP could affect tumor cells by inhibiting DNA methyltransferase [9], cellular kinases, modulating the MAPK signaling pathway [10]. NaVP shows antineoplastic activity based on its gene-regulation functions [11–13]; it has an effect on chloride, sodium ions transport in vivo [14], induces cell cycle arrest, and enhances the efficiency of glioma radiotherapy in clinical trials [15]. NaVP has been reported to have an anticancer effect on U87 cells at low dosages of the drug [8]. NaVP is able to induce apoptosis in glioma U87 cells in a dose-dependent manner through the activation of the mitochondria apoptosis pathway [16].

Further studies of GBM markers are needed to know how NaVP regulates tumor growth in experimental models. Polycomb group proteins (PRC1 and PRC2) regulate the chromatin structure and have an important regulatory role in human malignancies and catalyze histone (H2A and H3) modifications. Studies show the role of the PRC2 catalytic component enhancer of the zeste homolog 2 (EZH2) in neoplastic development [17]. EZH2 is actively involved in cell cycle progression, cell proliferation, differentiation, and apoptosis which are associated with human malignancy progression [17, 18]. EZH2 in glioblastoma leads to cell cycle arrest at the G_0_/G_1_ phase [19]. The EZH2 protein was found to be well expressed in U87 cell lines and its increased expression in human glioma tissue correlates with the glioma grade and a decreased GBM patient survival [20]. The EZH2 protein participates in mice embryo development [21]. EZH2 promotes the epithelial to mesenchymal transition program [22, 23]. EZH2 inhibitors have been an area of intense preclinical and clinical investigations and show a significant antitumor effect in various malignancies in animal models [24, 25].

The tumor suppressor gene* p53* is a cell cycle regulator protein associated with the suspension of cell growth and apoptosis induction [26]. Recently the p53 protein has been found to regulate cellular metabolism, stem cell function, invasion, metastases, and cell-cell communication within the tumor microenvironment [27]. Studies of* Trp53*^−/−^*Pten*^−/−^ mice showed that p53 promotes glioblastoma cells differentiation and inhibits the tumor development [28]. The tumor* p53* has a potential noncell autonomous function by modulating the expression of secreted proteins influencing the neighbor cells [29]. The loss of normal p53 function and the acquisition of oncogenic functions by mutant p53 proteins may contribute to tumorigenesis. The role of p53 in glioma progression is under ongoing discussion as the overexpression of mutated p53 may mark more aggressive tumor biology [30]. The expression of the protein p53 had a significant impact on the survival time: patients who did not have immunohistochemical expression of p53 had a significantly longer median survival time than those with its positive expression [31], but other researchers did not found opposite relationship [32]. The expression of p53 in the human glioma U87 cell line nuclei was found to depend on the used animal model: the percentage of human glioblastoma p53-positive nuclei of the same xenograft was higher in the tumors grown on the chick chorioallantoic membrane than in the brain of nude rat model [33].

The first-line treatment drug Temozolomide for GBM may only increase the survival of patients on average by a few months, and NaVP treatment sensitizes temozolomide-resistant glioma cells [8, 34]. Thus, it is urgently needed to develop novel strategies to increase the efficacy of the GBM treatment. The present study was designed to assess the human glioma cell U87 xenograft studied in the chicken embryo chorioallantoic membrane (CAM) model. CAM is widely used as an in vivo model for the investigation of tumor invasion, metastasis, and neoangiogenesis [35, 36]; also it is valuable model to study drug delivery systems [37]. CAM is a highly vascularized membrane located beneath egg shell and serves for metabolic exchange between air and chick embryo [38]. Chick embryo develops in 21 days until hatching and immune system matures completely only at the 18th embryo day development (EDD) [39]. The macroscopic tumor appearance, histopathology, and the immunohistochemistry of U87 cells untreated and treated with different NaVP concentrations as well as the expression of the selected p53 and EZH2 markers in xenograft tumor were tested.

The main objectives which indicate the novel and original aspects of the current study were to compare the evaluated characteristics of the tumor growth, penetration to the CAM mesenchyme, and correlation of these phenomena with the EZH2 and p53 expression in the tumor as a response to the treatment with different NaVP concentrations in the CAM model.

## 2. Materials and Methods

### 2.1. Egg Preparation for the Inoculation of Tumor Cells and the Study Groups

Fertilized chicken eggs* (Cobb 500)* were purchased from a local hatchery and incubated (Maino incubators, Oltrona S.M. (Co), Italy) for 7 days after breeding at 37°C and 60% humidity. For three consecutive days the eggs were rolled in an incubator once per hour. Then the eggs were cleaned with prewarmed 70% ethanol and a small hole was drilled in the location of an air sac. Approximately 2 ml of albumin was aspirated to create a false air sac directly over the CAM, allowing its dissociation from the egg shell membrane. Then a square window of approximately 1 cm^2^ was carefully drilled, opened, and sealed with sterile parafilm for the further inoculation of tumor cells. The eggs were then sealed with a transparent tape and returned back into the incubator until tumor cell grafting [40]. The cells were grafted on the 7th day of embryogenesis. Three fertilized chicken egg groups were studied: (1) nontreated (control) (*n* = 10), (2) treated with a 4 mM of NaVP (*n* = 10), and (3) treated with 8 mM of NaVP (*n* = 10).

### 2.2. U87 Cell Placement on the CAM

Commercial human glioblastoma U87 cells line was obtained from the Institute of Neuroscience (Kaunas, Lithuania) and kept in Dulbecco's modified Eagles medium (DMEM) (Gibco, USA) supplemented with 10% fetal bovine serum (Gibco, USA) and with 100 IU/mL of penicillin and 100 *µ*g/mL streptomycin (Gibco, USA). The amount of 1 × 10^6^ U87 cells was resuspended in 10 *µ*l of the DMEN (1x) + GlutaMAX (GIBCO, USA). These cells (in 10 *µ*l of the medium) were mixed with 10 *µ*l type I rat tail collagen (Gibco, USA) commonly used to form visible tumors on the chicken embryo chorioallantoic membrane. The total amount of 20 *µ*l of the mixture was then dropped onto an absorbable surgical sponge (Surgispon®) which was cut by hand with a blade into equal pieces of 9 mm^3^ (3 × 3 × 1 mm). Each piece of the sponge (one per embryo) was later gently placed on the top of the growing CAM on the day 7 (EDD7) of embryo development (*n* = 30).

The NaVP-treated and control specimens were collected after 5 days of incubation on the 12 days of embryo development (EDD12), fixed in a buffered 10% formalin solution for 24 h and then paraffin-embedded.

### 2.3. Haematoxylin and Eosin (H–E) Staining

Each embryo of different experimental groups was sacrificed and a CAM was removed, fixed in 10% neutral-buffered formalin, dehydrated, and embedded in paraffin. Serial sections of 3 *μ*m were cut and stained using the standard H–E technique. After overnight incubation at 37°C the sections were deparaffinized in xylene, dehydrated in graded series of ethanol (70%, 90%, and 96%), stained with H–E, and then cleared with xylene and mounted using a mounting medium (Roti®-Histokitt II, Germany).

### 2.4. Biomicroscopy In Vivo and Light Microscopy for the Visualization of Formed Tumors In Vivo

CAMs with grafted U87 cells were registered in vivo daily under a stereomicroscope (SZX2-RFA16, Japan) equipped with an Olympus DP72 camera for both video recordings and acquiring still images. CAMs were investigated from day 2 (EDD9) after grafting to day 5 (EDD12). Histological slides were investigated under a light microscope Olympus BX40F4 (*Olympus Optical Co. Ltd.*, Japan) and photographed with an Olympus digital camera (XC30, Japan) using CellSens Dimension 1.9 Digital Imaging Software.

### 2.5. Immunohistochemistry and Cell Count

Paraffin blocks of CAM with grafted tumors were cut into 3 *µ*m slices and then processed using standard deparaffinization and rehydration techniques. The polyclonal anti-KMT6/EZH2 (phospho S21, ab84989, Abcam) and monoclonal anti-p53 (aa 211-220, clone240, CBL404, Millipore) antibodies were used as the primary antibodies to detect positively stained U87 cells. The primary antibody was detected using biotinylated secondary antibody (DAKO EnVision Flex + Mouse) followed by horseradish peroxidase-conjugated streptavidin (DAKO EnVision Flex/HRP) used as recommended by the manufacturer. Finally, positive reactions were visualized using a 3,3′-diaminobenzidine chromogen (DAB, DAKO, Glostrup, Denmark). After incubation in chromogen, the slides were counterstained with haematoxylin, dehydrated, cleared in xylene, and mounted with a mounting medium. In every formed tumor, EZH2 and p53 positive cells were counted. In every tumor, two equal fields were randomly selected (size of the field 10000 *µ*m^2^). In every field, all cells were counted, positively stained cells and calculated percentage of EZH2 and p53 cells. Data were presented as % of positively stained cells in every group.

### 2.6. Investigation of Tumor Invasion into Chorionic Epithelium and Mesenchyme

Serial histological sections of the experimental tumors and CAM were performed to evaluate tumor invasion. According to tumor behavior on the membrane they were distributed into three groups: (1) tumor was formed on the surface of the CAM, chorionic epithelium was not destroyed, and there was no invasion into the mesenchyme; (2) chorionic epithelium was destroyed and cells invaded the mesenchyme, but part of the tumor remained on the membrane surface; (3) tumor cells destroyed the chorionic epithelium, invaded the mesenchyme, and were completely surrounded by the mesenchyme.

### 2.7. Statistical Analysis

Data presented as mean and standard deviation. Data were compared using Tukey's test applying one-way ANOVA. Statistical package SPSS 20.0 was used. Difference was considered as significant when* p* < 0.05. For visualization of the data, Sigma Plot 11.0 program was used. Data on cells positively stained for the EZH2 and p53 count were tested for normality distribution using two tests: the Kolmogorov–Smirnov and the Shapiro–Wilk (the Shapiro–Wilk test is more suitable for small-size samples). Both tests showed the normality of data distribution, and one-way ANOVA was applied for the data analysis.

## 3. Results

### 3.1. Biomicroscopy Data In Vivo

**Figure 1:**
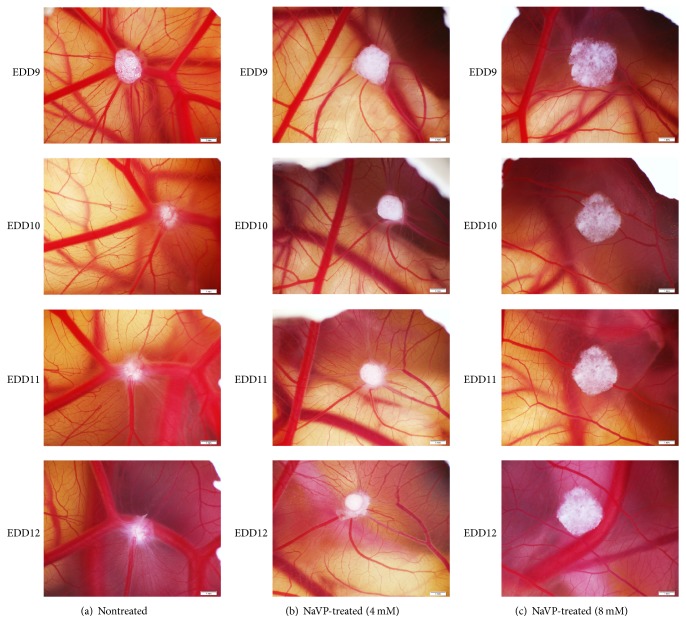
Tumor xenograft on CAM of nontreated and NaVP-treated U87 cells during days 9–12 of embryo development. Figure 1 represents the U87 cell tumor xenografts on CAM growth dynamics, which was photographed daily on days 9–12 of embryo development (EDD9–12): (a) represents nontreated tumors, (b) NaVP-treated (with 4 mM of NaVP) tumors, and (c) NaVP-treated (with 8 mM of NaVP) tumors. The clearness of expression of nontreated tumor edges from the EDD10 gradually diminishes. This is related to tumor cell penetration into the mesenchyme since the EDD10 and with a clear and fast U87 cell invagination in the mesenchyme (a). Around nontreated tumors, the spoked-wheel pattern is clearly expressed in the CAM on day EDD12 (a), while on the same day in the 4 mM and 8 mM NaVP-treated grafted tumors they failed to attract new blood vessels ((b) and (c), resp.). In the pictures of (c), the size of tumors treated with 8 mM of NaVP in EDD9–12 is more pronounced as compared with tumors in (b), which is related to tumor cell penetration into the mesenchyme after treatment with 4 mM of NaVP. In the pictures of (c), the spoked-wheel patterns are practically absent around the tumors. Scale bar: 1 mm.

**Figure 2:**
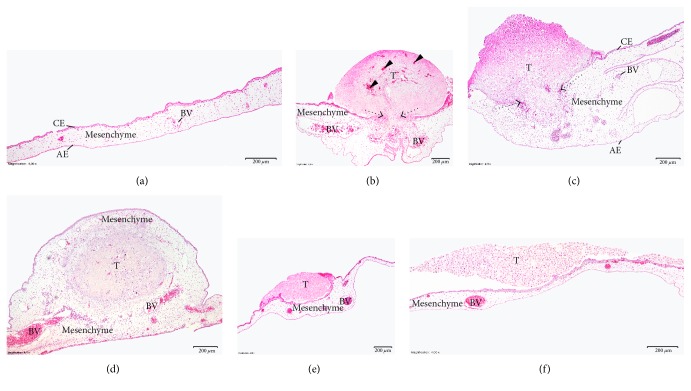
The CAM with U87 cell tumor at day 12 of embryo development. CE: chorionic epithelium, AE: allantoic epithelium, BV: blood vessels, and T: tumor. (a) Control membrane without tumor. (b) Membrane with tumor of U87 cells without treatment. Arrowheads show the growth of chicken blood vessels into the tumor, and a dotted arrow shows the chorionic epithelium destroyed by tumor cells. (c) Membrane with tumor developed from U87 cells treated with 4 mM NaVP. The dotted arrow shows the destroyed chorionic epithelium and partial tumor invasion into the mesenchyme. (d) Membrane with a tumor developed from U87 cells without treatment. The tumor is completely encapsulated into the CAM mesenchyme. (e) Membrane with tumor developed from cells treated with 4 mM of NaVP, with the intact chorionic epithelium; the tumor is not vascularized and located on the surface of the CAM. (f) Tumor developed from cells treated with 8 mM of NaVP, located on the surface of the membrane, not vascularized. Scale bar: 200 *μ*m.

The U87 cells tumor xenografts inoculated on egg CAM and photographed daily via the shell window on days 9–12 of embryo development (EDD9–12) are shown in Figure 1.  Figure 1(a) shows the development dynamics of nontreated U87 cell tumors during EDD9–12. In this group, the in vivo biomicroscopy highlighted the penetration of tumors into the underlying mesenchyme starting from day 2 after inoculation (at EDD9). Nontreated tumors are seen surrounded by formed new blood vessels and a clearly expressed spoked-wheel pattern which was observed on days 4 and 5 after grafting (Figure 1; EDD11-12). When cells were treated with 4 mM of NaVP, they formed condensed tumors on CAM without invagination into the chorionic mesenchyme (Figure 1(b), EDD10–12). The tumors that developed from 8 mM of NaVP-treated U87 cells did not show clear dynamics during 5 days of development: the in vivo biomicroscopy tumor images are very similar during days 2–5 (Figure 1(c), EDD9–12), and tumors failed to attract blood vessels. The 8 mM NaVP-treated tumor cells were found distributed on CAM without penetrating the membrane. Such distribution of cells on the surface of CAM produces the misleading impression that the tumors have increased in size (Figures 1(c) and 2).

### 3.2. Histological Investigation of CAM with Implanted Tumors

Images of H–E-stained slides of control CAM, the CAM with nontreated U87 cell tumors, and CAM with tumors of U87 cells treated with 4 mM and 8 mM NaVP are presented in Figure 2. The control CAM is a thin membrane with the developed chorionic epithelium, allantoic epithelium, and a mesenchyme between these two epithelial layers and blood vessels (Figure 2(a)). The U87 cells form tumors on the CAM, which are related to the clearly thickened CAM mesenchyme under the onplant of nontreated tumors (Figures 2(b) and 2(c)) or the tumor is encapsulated in the thickened CAM mesenchyme (Figure 2(d)). The nontreated tumors are vascularized; blood vessels with chicken blood are clearly visible in them. The chorionic epithelium under the onplant is destroyed in most cases, and the invasion of glioblastoma cells into the CAM mesenchyme is obvious (Figures 2(b) and 2(c)). Tumors developed from U87 cells treated with 4 mM and 8 mM of NaVP are not vascularized, the chorionic epithelium is intact, glioblastoma cells are distributed on the CAM surface, and such distribution is more pronounced in tumors treated with 8 mM of NaVP (Figures 2(e) and 2(f), resp.). The nontreated tumor causes mesenchyme hyperplasia which is reduced by the treatment with NaVP, and this depends on the NaVP dose (Figure 2).

### 3.3. Tumor Invasion into Chorionic Epithelium and Chorioallantoic Membrane Mesenchyme

**Figure 3:**
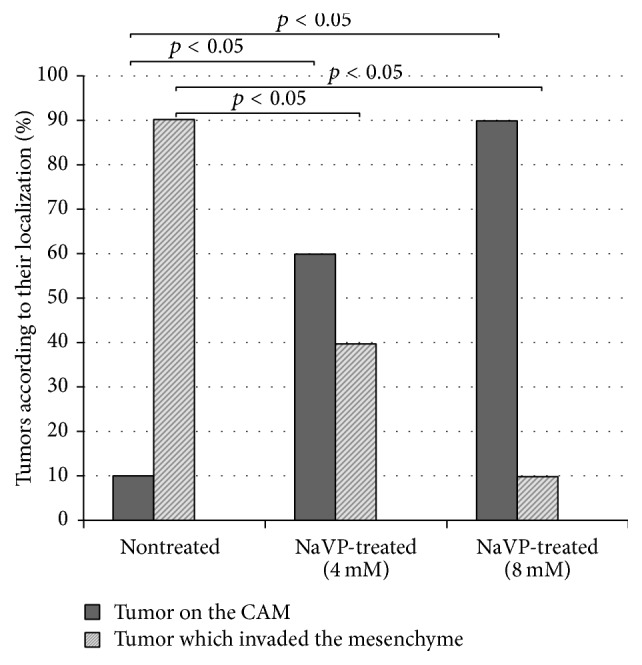
Frequency of U87 cell tumor groups with invasion into the CAM mesenchyme and localized only on the CAM. The investigated groups were U87 cell tumors nontreated with NaVP (*n* = 10), treated with 4 mM of NaVP (*n* = 10), and treated with 8 mM of NaVP (*n* = 10). Tumors invading the CAM mesenchyme are presented as the common group containing tumors of which part of tumor invaded the mesenchyme and those localized only in the mesenchyme.

Of the tumors developed from nontreated U87 cells, 50% destroyed the chorionic epithelium, invaded the chorioallantoic membrane mesenchyme, and had been completely formed in the mesenchyme; 40% of the tumors destroyed the chorionic epithelium and invaded the mesenchyme but partly remained on the CAM surface, and only 10% of tumors were formed on the surface of the CAM without invading the mesenchyme. When U87 cells were treated with 4 mM of NaVP, 60% of tumors were formed on the surface of the CAM and 30% destroyed the chorionic epithelium and invaded the mesenchyme, but part of the tumor remained on the surface of the CAM. In this group, 10% of tumors were observed only in the mesenchymal layer of the CAM. When U87 cells were treated with 8 mM of NaVP, 90% of the tumors were formed on the surface of the CAM without invading the mesenchymal layer, the chorionic epithelium was also not destroyed, and only 10% of cases showed a complete invasion into the mesenchyme. Compared with nontreated tumors, the incidence of tumor formation on the CAM surface without invading the mesenchyme was significantly higher when U87 cells were treated with 4 mM and 8 mM of NaVP (10%, 40%, and 90%, resp.;* p* < 0.05). Comparing the incidence of the nontreated tumors and tumors whose U87 cells had been treated with 4 mM and 8 mM of NaVP, tumors invading the CAM mesenchyme (the common group of tumors of which part invaded the mesenchyme and those localized only in the mesenchyme) were found significantly less frequently in the NaVP-treated tumor groups as compared with nontreated tumors (10%, 40%, and 90%, resp.;* p* < 0.05). Tumors on the CAM were found more frequently in the 4 mM NaVP-treated group as compared with the nontreated tumor group (*p* < 0.05), and comparing nontreated and the 4 mM NaVP-treated groups, the tumors which invaded the mesenchyme were significantly more frequent in the nontreated groups of tumor (*p* < 0.05; Figure 3).

### 3.4. Immunohistochemical Investigation 

#### 3.4.1. The EZH2 Protein Expression

**Figure 4:**
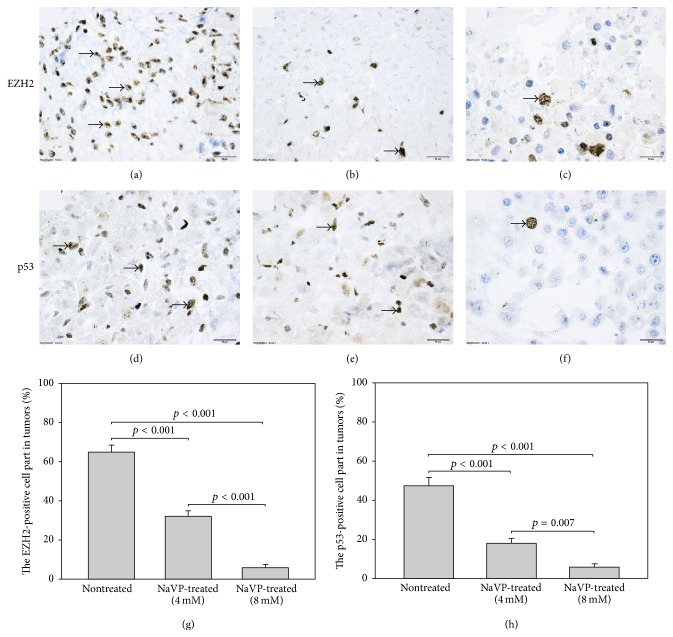
Expression of EZH2 and p53 proteins in tumors formed by U87 cells treated with 4 mM and 8 mM of NaVP and by nontreated cells. (a) EZH2 expression in nontreated tumors, (b) EZH2 expression in tumors treated with 4 mM of NaVP, (c) EZH2 protein expression in tumors treated with 8 mM of NaVP. (d) p53 protein expression in tumors formed by nontreated U87 cells, (e) p53 expression in tumors treated with 4 mM of NaVP, and (f) p53 protein expression in tumors treated with 8 mM of NaVP. (g) EZH2-positive cells and (h) p53-positive cell tumors developed from U87 cells in tumor groups without treatment and from cells treated with 4 mM and 8 mM of NaVP, respectively. Arrows indicate EZH2- and p53-positive cells.

In tumors formed by the U87 cells without treatment a high expression of the EZH2 protein was found, and positively stained cells were distributed in all tumor (Figure 4(a)). The EZH2 protein was expressed exclusively in the nuclei of the tumor cells. In tumors treated with 4 mM of NaVP, cells with a positive staining for the EZH2 protein were located mostly in the upper part of a tumor (Figure 4(b)). In tumors treated with 8 mM of NaVP, a weak staining for the EZH2 protein was observed (Figure 4(c)). The positive expression of the EZH2 protein was observed also in the chorionic and allantoic epithelium and CAM mesenchyme in all investigated experimental groups. The EZH2-positive cell count in tumors formed by the nontreated U87 cells was 65 ± 13% of all tumor cells. The EZH2-positive cells made 32 ± 12% of all cells in tumors formed by cells treated with 4 mM of NaVP. In tumors treated with 8 mM of NaVP, only 6.3 ± 5% of all tumor cells were positively stained for the EZH2 protein. The number of EZH2-positive cells was significantly lower in cells treated with 4 mM and 8 mM of NaVP as compared with the cell count in tumors formed by cells without treatment (*p* < 0.001; Figure 4(g)), and the expression of the EZH2 protein was significantly higher in 4 mM NaVP-treated group tumors as compared with the 8 mM of NaVP-treated ones (*p* < 0.001; Figure 4(g)). Scale bar: 20 *μ*m.

#### 3.4.2. The p53 Protein Expression

In tumors formed by nontreated U87 cells, a high expression of the mutant p53 protein was found (Figure 4(d)). In tumors treated with 4 mM of NaVP, the number of p53-positive cells diminished, and in tumors treated with 8 mM of NaVP only a small number of positively stained cells were observed (Figures 4(e) and 4(f)). The p53-positive cell count in tumors without treatment was 47.3 ± 13% of all tumor cells. In tumors treated with 4 mM of NaVP, the p53-positive cells made 18 ± 8% of all cells, and in tumors treated with 8 mM of NaVP only 6.4 ± 5% of all tumor cells were positively stained for the p53 protein. The number of p53-positive cells was significantly lower in cells treated with 4 mM and 8 mM of NaVP as compared with the cell count of nontreated tumors (*p* < 0.001), and treatment with 8 mM of NaVP significantly diminished the expression of p53 as compared with 4 mM of NaVP-treated tumors (*p* < 0.007; Figure 4(h)). Scale bar: 20 *μ*m.

## 4. Discussion

GBM is the most lethal form of cancer with a median survival of up to 12 months [1]. Seizures occur in up to 90% of patients with low-grade gliomas and in up to 60% with high-grade gliomas [41, 42]. Tumor growth stimulates seizures, and seizures activate tumor growth [43]. The antiepileptic drug NaVP has the promising anticancer effects: it stimulates histone acetylation, leading to an unfolding of the chromatin structure that leaves DNA more susceptible to the effects of chemotherapy and radiation therapy [44]. In vitro and in vivo combined NaVP and temozolomide treatment induces apoptosis and autophagy of cancer cells [45, 46]. The EORTC trial of temozolomide and chemoradiation showed that the median survival of glioblastoma patients who received NaVP was significantly longer than of patients treated by chemoradiation alone [47, 48].

In vivo experimental studies demonstrate that the CAM model allows a successful testing of investigational medicinal products. The CAM model has been used to implant several malignant cell lines to investigate tumor growth and the metastatic process, angiogenic potential, identifying therapeutic targets, and evaluating antitumor drugs, as these transplanted cells on CAM keep producing human antigens [33, 34, 49]. CAM is formed on days 4 to 5 of embryo development. The CAM is a thin, highly vascularized membrane located beneath the egg shell and consisting of the chorionic epithelium, mesenchyme, and allantoic epithelium [36]. A chick embryo until day 18 of EDD may serve as a naturally immunodeficient host for the investigation of transplanted cells [38, 50]. Investigators suggest that experimental glioma growth on the CAM exhibits a sufficient similarity with fundamental aspects of the human disease: defined tumor growth with key features of human glioblastoma at cellular and molecular levels occurs in a reproducible manner after human GBM cell grafting [51]. GBM is characterized by a fast cell proliferation, infiltrative migration, and the angiogenesis induction [20, 52].

The study biomicroscopy follow-up of control-nontreated and NaVP-treated U87 cell tumors on CAM shows a progressive growth and vascularization. The majority of tumors which developed from U87 cells without NaVP treatment during tumor growth destroyed the chorionic epithelium and invaded the mesenchyme. Biomicroscopy showed that the nontreated xenograft of U87 cells on CAM invaginated into the underlying mesenchyme starting from day 2 after inoculation. The study shows that increasing the NaVP concentration diminished the U87 cell capability to destroy the chorionic epithelium and significantly increased the number of tumors formed only on the surface of the CAM without invading the mesenchyme. The incidence of tumor formation on CAM without invasion into the mesenchyme was significantly higher when U87 cells have been treated with NaVP, and this effect significantly increased with the NaVP concentration. NaVP is a histone-deacetylase (HDCE) inhibitor and specifically inhibits HDAC classes I and IIa [53]. NaVP is able to induce apoptosis in glioma U87 cells in a dose-dependent manner [16], and the U87 cells may be more sensitive to NaVP than the other GBM lines [8]. Other researchers investigation of NaVP effects on tumor cells in vitro demonstrated that it inhibited cell proliferation by causing cell cycle arrest in the G1 and/or G2 phase and that it induced differentiation and/or apoptosis in various cancer cells [54, 55], reduced the proliferation of glioblastoma-derived stem cells [56], and decreased the viability of primary human glioblastoma cells [57].

The U87 tumors on CAM in relationship with tumor progression concomitantly induced blood vessel angiogenesis, and a clearly expressed spoked-wheel pattern was observed on days 4 and 5 after grafting in nontreated tumors. The treatment of U87 cells with 8 mM of NaVP did not show clear dynamics of tumor growth during 5 days of development at EDD9–12; at the same time, the blood vessel angiogenesis failed. Other researchers reported that the progression of the U78 tumor was related to angiogenesis which indicates a glioma development relationship with growth factor receptors [58], and platelet-derived growth factor receptors [59] are important for angiogenesis and glioma progression, and their inhibition could result in the inhibition of experimental glioma growth [51].

The studied GBM U87 cell tumor samples showed a strong nuclear staining of EZH2 in U87 cell tumors without NaVP treatment, and a high expression of the EZH2 protein in the tumor cell nuclei was found. It is known that increased EZH2 expression correlates with glioma grade and its recurrence, suggesting that EZH2 could be a marker of glioma aggressiveness and correlate with a decreased GBM patient survival, and the EZH2 protein was found to be strongly expressed in U87 cell lines. EZH2 is present only in dividing cells [20].

In our study, the expression of EZH2 protein was observed also in the chorionic and allantoic epithelium and CAM mesenchyme in nontreated with NaVP tumors. This positive staining may be associated with the fact that the EZH2 protein participates in embryo development: the EZH2 knockdown was shown to be embryo-lethal in mice [21]; the knockdown of EZH2 in cancer cells resulted in diminished tumor growth and reduced metastases in vivo [53, 60, 61]. EZH2 in glioblastoma leads to cell cycle arrest at the G_0_/G_1_ phase, further leading to the uncontrolled cell cycle progression in glioblastoma cells [19]. EZH2 is actively involved in cell cycle progression, cell proliferation, cell differentiation, and apoptosis [62]. EZH2 promotes the epithelial to mesenchymal transition program which is a known mechanism inducing tumor aggressiveness and metastases [22, 23].

We found that the number of EZH2-positive cells was significantly lower in tumors treated with 4 mM and 8 mM of NaVP as compared with the cell count in nontreated tumors, and the NaVP effect was higher as its concentration increased to 8 mM. It was reported that the inhibition of EZH2 may be a potential therapeutic strategy to target GBM proliferation, migration, and angiogenesis as the inhibition of EZH2 in vitro by pre-miR-101, EZH2 siRNA, or small molecule DZNep attenuated GBM cell growth, migration/invasion, and GBM-induced endothelial tubule formation in a U87-Fluc-mCherry GBM xenograft mouse imaging model resulted in a reduced tumor growth and migration/invasion. A significant correlation between the expression of 28 out of 279 genes associated with cell migration and EZH2 expression was observed [20]. The effectiveness of NaVP on EZH2 in U87 tumors indicates that NaVP has a potential therapeutic strategy to target GBM proliferation, invasiveness, and differentiation. Furthermore, several studies have reported that EZH2 inhibition increases the sensitivity of different cancer cells to radiation and chemotherapy [63–65]. The inhibition of EZH2 in B cell lymphomas induces p53-mediated apoptosis under DNA damage accumulation, resensitizing lymphomas cells to chemotherapy [66].

In the nontreated U87 cell tumors, the EZH2 expression in cells has been very strongly expressed in tumor areas which invaded across the chorionic membrane to the mesenchyme and were surrounded by the thickened mesenchyme. In this respect, of significance could be the results of other studies indicating that tumor infiltrating front, molecular heterogeneity in GBM can improve the rationale of potential molecular targets. The key genes involved in gliomas tumor cell proliferation, invasion, migration, response to immune system, and stemness markers are highly enriched in the peritumoral brain zone, and these genes probably contribute to the resistance of cells to standard therapy, resulting in a tumor recurrence [67].

The study shows that, in tumors formed by the U87 cells without NaVP treatment, a high expression of the p53 protein was found. The p53 is a tumor suppressor gene implicated in the genesis of malignancies. The overexpression of the p53 protein is often used as a surrogate indicator of mutations in the p53 gene [68]. The role of p53 in glioma progression is under ongoing discussion, because the overexpression of mutated p53 may mark the biology of a higher tumor progression [30]. The p53 protein is a transcription factor important in tumor growth, cell apoptosis [69, 70], activating the cell cycle [70], differentiation [71], and being involved in DNA repair [72]. The p53 gene encodes a 393-amino acid protein; p53 is a phosphoprotein that resides in the nucleus. The p53 gene mutations are one of the distinct features seen in GBM [73]. The nuclear overexpression of p53 in secondary GBM reflects the presence of mutant p53, and these mutations are involved in GBM progression [74]. However, the incidence of p53 protein accumulation in glioma is more frequently seen than p53 mutations [75–77] and the percentage of glioma cells, in which p53 protein accumulation is found to increase from the first biopsy to recurrent tumors [76]. Furthermore, p53 expression in GBM may accumulate in the cytoplasm. The role of p53 in cytoplasmic location is not clear. Some researchers indicate that it could be inactive [30, 75]. The expression of p53 in the human glioma U87 cell line nuclei was found to depend on the used animal model: the percentage of human glioblastoma p53-positive nuclei of the same xenograft was higher in the tumors grown on the chick chorioallantoic membrane than in the brain of a nude rat model [33]. Furthermore, the historical focus on p53 in the nucleus is broadened with its localization in mitochondria [78].

The p53 abnormalities are common in the progression from a low-grade lesion to a high-grade lesion of GBM in patients [73, 79], and the association between p53 mutations and GBM progression remains unclear [30]. The status of p53 had a little effect on the survival of GBM patients [74, 80]. Some reported that p53 expression was more common in the long-term survival irrespective of the specific types of p53 mutation [32], while others, on the contrary, indicated that patients who did not have immunohistochemical expression of p53 had a significantly longer median survival time than those with its positive expression [31]. No relation of the p53 status and time with tumor progression was found [81]. Several studies have indicated that p53 was significantly altered in patients with malignant transformation rather than in those with no apparent progression [82, 83]. Also, wt p53 failed to sensitize glioma cells to cytotoxic drugs and, therefore, contributed to chemoresistance [84].

Our study revealed a high expression of p53 in U87 cell tumors which were not treated with NaVP, and this expression was accompanied with pronounced angiogenesis in tumors in the CAM model. The p53 distribution in the tumor was not related to tumor periphery or vascularization areas. Other investigators also found that the pattern of p53 expression was not related to a particular region, such as the infiltrating edges or vascularization areas in GBM of patients [85]. Others reported that the cytoplasmic p53 expression was exclusively seen in the perivascular areas in 57% of de novo cases of GBM, and the expression of cytoplasmic p53 around the perivascular area may represent its role in angiogenesis [30]. Immunoreactivity of the vascular endothelial growth factor (VEGF) in GBM cells was associated with vascularity and positively correlated with p53 expression, suggesting an association between mutant p53 and VEGF [86].

In our U78 tumors on CAM treated with NaVP, the number of p53-positive cells significantly diminished, and only a significantly decreased number of p53-positive cells in tumors treated with 8 mM of NaVP were observed. By others, the mechanistic insight shows that p53 directly interacts with the antiapoptotic proteins bcl-xL and bcl-2, and mutant p53 proteins could be related to this binding [87]. It was reported that NaVP induced by p53-dependent mitochondrial localization of Bax and Bcl-xL, as well as the mitochondrial membrane potential and cytochrome c release, are important for the p53 role in NaVP-mediated radiosensitization of cancer cells [88].

## 5. Conclusion

The study results demonstrate that the CAM model allows a successful testing of anticancer drugs designed to interfere with the p53 and EZH2 molecular pathways important for glioma progression. The experimental findings of the study indicate that NaVP has a function in blocking the proliferation, migration, and angiogenesis of human U87 glioma cells in the CAM tumor model, thereby supporting the NaVP potential in glioblastoma therapy. However, the U87 cell model system used in the study has limitations due to the known high cell line mutability and variability, but the study results are important for further studies to evaluate the NaVP effect on the p53 and EZH2 expression of different (pediatric and adult) cell and brain tumor stem cell lines as well as the primary GMB tumor cell cultures.
